# Role of *Lactobacillus reuteri* *DSM 17938* on Crying Time Reduction in Infantile Colic and Its Impact on Maternal Depression: A Real-Life Clinic-Based Study

**DOI:** 10.3390/clinpract12010005

**Published:** 2022-01-07

**Authors:** Arun Wadhwa, Dhanasekhar Kesavelu, Kishore Kumar, Pallab Chatterjee, Pramod Jog, Sarath Gopalan, Rudra Paul, Krishna Chaitanya Veligandla, Suyog Mehta, Amey Mane, Sucheta Pandit, Rahul Rathod, Sushma Jayan, Monjori Mitra

**Affiliations:** 1 Dr. Wadhwa’s Clinic, New Delhi 110048, India; arun@drwadhwasclinic.com; 2 SS Child Care, Chennai 600037, Tamil Nadu, India; drdskgastro@gmail.com; 3 Cloudnine Hospital, Bangalore 560066, Karnataka, India; drkishore@cloudninecare.com; 4 Outreach Clinic, Clinical and Experimental Pharmacology, School of Tropical Medicine, Kolkata 700073, West Bengal, India; pallabchatterjee@gmail.com (P.C.); rdrpaul@gmail.com (R.P.); 5 Medipoint Hospital, Pune 411007, Maharastra, India; dr_pramodjog@yahoo.co.in; 6 Madhukar Rainbow Children’s Hospital, New Delhi 110017, India; sarathgopalan@gmail.com; 7 Global Generics India, Dr. Reddy’s Laboratories Limited, Hyderabad 500016, Telangana, India; krishnacv@drreddys.com (K.C.V.); drsuyogmehta@gmail.com (S.M.); amey.mane@drreddys.com (A.M.); Suchetaakshay.p@drreddys.com (S.P.); rahul.rathod@drreddys.com (R.R.); 8 Medclin Research Pvt. Ltd., Kolkata 700107, West Bengal, India; sushma.jayan@medclinsearch.com; 9 Institute of Child Health, Kolkata 700017, West Bengal, India

**Keywords:** infantile colic, excessive crying, crying duration, maternal depression, fussiness, *Lactobacillus* *reuteri DSM 17938*

## Abstract

**Simple Summary:**

*Lactobacillus* *reuteri DSM 17938* is beneficial for reduction of crying time in infantile colic, as demonstrated through multiple randomized controlled clinical trials. This clinic-based real-life study is the first to evaluate the role of *L. reuteri DSM 17938* in infantile colic in India. We observed that *L. reuteri DSM 17938* supplementation in infantile colic subjects resulted in a significant reduction of crying time and unexplained fussiness. A significant number of subjects reported 50% reduction in crying time throughout the study duration. There was also a significant reduction in maternal depression scores, from baseline to study end.

**Abstract:**

Background: Infantile colic is characterized by prolonged periods of inconsolable, incessant crying and persistent fussing in an otherwise healthy infant. It is a self-limiting condition, but causes significant stress to mothers. AIM: To observe the role of *Lactobacillus reuteri* *DSM 17938* in reducing crying time in colicky infants in routine clinical practice. Methods: This was a prospective observational multicentric clinic-based study. Each practitioner included approximately 30 infants < 5 months of age with infantile colic who were prescribed *L. reuteri* *DSM 17938* for a period of 21 days. There were four physical consultations and two telephonic consultations. The parents were given a daily diary to record the duration of crying and fussing episodes and a questionnaire was administered during the consultations. Results: A total of 120 infants with a mean age of 56.9 ± 34.2 days were included in this 28-day study. The mean crying time as reported by the parents in the subject diary reduced from 248.2 ± 101.2 min, 95% CI: 229.45, 266.94 at baseline to 45.6 ± 79.1 min 95% CI: 31.02, 60.31 at study end (*P* < 0.01). The clinical response (defined as reduction of 50% in crying time) was observed in 85% of subjects at study end. The fussiness and parental perception of colic recorded during the consultations were reduced by 66% and 72%, respectively, at study end. The maternal depression scores were reduced to 63% at study end. Conclusion: *L. reuteri DSM 17938* was associated with a significant reduction in crying time in colicky infants, and showed improvement in maternal depression.

## 1. Introduction

Infantile colic is defined as recurrent prolonged periods of irritability, fussing, or crying in infants reported by parents, and occurring without any obvious cause. Physiologically, infantile colic is characterized as a functional gastrointestinal disorder, with recurrent gastrointestinal symptoms, but without any notable structural or biochemical abnormality. The updated ROME IV guidelines delineate the condition’s related definitions for daily clinical practice and clinical research [1]. According to a literature review in 2016, infantile colic is estimated to occur in about 20% of all infants [2]. 

*Lactobacillus reuteri DSM 17938* is a widely researched probiotic that is an effective and safe supplement available for use in the management of infantile colic. Numerous placebo-controlled studies have demonstrated that administration of *L. reuteri DSM 17938* can significantly reduce crying time in infants with colic, and in such the response rates of having a 50% or greater decrease in crying/fussing time were 2.3-fold greater compared to controls [3,4,5]. However, Sung et al. [6], in their randomized controlled trial on 167 infants, found no benefit with *L. reuteri* supplementation in colicky infants, and the authors recommended further research to establish positive outcomes associated with probiotic use in infantile colic. In contrast, a recent meta-analysis by Sung et al. [7] published in 2018 suggested that administration of *L. reuteri DSM 17938* can significantly reduce crying time and cause the supplemented children to be almost twice as likely as those in the placebo group to experience treatment success at all time points. Numerous studies have also evaluated the association of infantile colic and maternal depression that results in increased risk of maternal anxiety, child abuse, and early cessation of breastfeeding [8,9].

In India, there is lack of data on *L. reuteri DSM 17938* for crying time reduction in infantile colic and also the subsequent consequences like maternal depression. Considering the change in practice in the management of infantile colic with the availability of *L. reuteri DSM 17938* in India, a real-life study was planned in clinics spread across geographical zones. The aim was to evaluate the role of *L. reuteri DSM 17938* along with standard of care, as appropriate, on the reduction of crying time and fussiness and its impact on parents’ perception of colic and maternal depression.

## 2. Materials and Methods

### 2.1. Study Design and Setting

This was a prospective, observational, multicentric study conducted across zonal clinics in India.

The sample size was calculated based on the incidence rate of colic assumed to be around 20%. With α  =  0.05, 97 subjects were to be enrolled and to allow for a 20% dropout rate, a total of 120 subjects are to enrolled in the study. A total of 120 infants diagnosed with infantile colic and prescribed *L. reuteri DSM 17398* were enrolled between 3 January 2019 to 3 January 2020 from the outpatient departments of clinics and institutions in the cities of New Delhi, Kolkata, Pune, and Bangalore (Institute of Tropical Medicine, Kolkata, Medipoint Hospital, Pune, Cloudnine Bangalore, SS Child Care Chennai, Wadhwa’s Clinic, New Delhi & Madhukar Rainbow Children’s Hospital, New Delhi). Written informed consent was provided by the parents. The parents were provided a daily diary to record the duration of crying episodes, the medications given, and any adverse events. Questionnaires were provided during in-clinic consultations to record the maternal depression scores and parental perceptions of colic severity and fussiness.

The study included four physicals, conducted during day 1, 7, 21, and 28 consultations, and two telephonic consultations, conducted on day 3 and day 14. The demographic details and crying and fussing history of the infants were recorded during inclusion at day 1. Parents were requested to visit the study site for submitting the daily diary card and fill-in the questionnaires. The day 3 and day 14 telephonic consultations were conducted to evaluate the infant’s colic symptoms.

### 2.2. Study Participants

Infants of either sex, <5 months of age, who were either exclusively or predominantly (>50%) breast fed, diagnosed with infantile colic, and prescribed 5 drops once daily of *L. reuteri DSM 17398* with 10^8^ CFUs for a period of 21 days were eligible for study participation. The enrolled infants were followed up for an additional 7 days after treatment completion. Infants with significant co-existing medical illnesses, any other gastrointestinal disease, failure to thrive or who were on antibiotics probiotics or acid suppressive therapy for a period of 2 weeks before the start of study were ineligible for study enrollment. The study protocol was approved by the ethics committees of all participating clinics (The Institutional Ethics Committee, Cloudnine Hospital, Bangalore, Good Society for Ethical Research, New Delhi, Penta-Med Ethics Committee, Pune, Clinical Research Ethics Committee, Calcutta School of Tropical Medicine, Kolkata and Ripon Independent Ethics Committee, Chennai, India). The study was registered with the clinical trials registry of India (CTRI/2018/12/016743) and was conducted over a period of 1 year, from January 2019 to January 2020.

### 2.3. Study Outcomes and Assessments

The primary objective of the current study was to evaluate the reduction in crying time of the treated infants and the clinical response (defined as the percentage of infants achieving a reduction in the daily average crying time > 50% during the study period). The secondary objective was to evaluate the impact on maternal depression with reduction in crying time and infants’ fussiness and parental perception of colic severity.

The primary outcomes were to assess the reduction in daily crying time from baseline to end of the study and to measure the clinical response, defined as the percentage of responders with > 50% of reduction in crying time during the study period compared to baseline. The secondary outcomes were recorded at day 0, 7, 14, 21, and 28, and included maternal depression as assessed by Edinburgh’s postnatal depression scale (commonly referred to as EPDS) [10]. The scale consisted of 10 questions, with a maximum score of 30. Mothers whose score was 13 or above were likely to be suffering from depression. Fussiness was recorded on a Fussiness Rating Scale (commonly referred to as FRS) with the total fussing duration (in h), amount and intensity of fussiness scored on a scale of 0–6 (0: No fussiness to 6: Constant fussiness) [11]. To assess the parental perception of colic, a 0–10 cm visual analogue scale (VAS) was used, with 0 indicating no pain and 10 indicating the worst pain.

### 2.4. Adverse Events

Parents were instructed to inform the investigator of any adverse events that occurred during the study and to record it in the daily diary.

### 2.5. Statistical Analysis

The statistical analysis was conducted using International Business Machines Corporation (IBM) Statistical product and service solutions (SPSS) v.26, 2019, IBM Corp. (Armonk, NY, USA) Continuous variables with underlying normal distribution were compared using suitable parametric tests, such as *t*-tests or analysis of variance. Non-continuous and non-normal data were tested using the non-parametric Wilcoxon test.

#### Exploratory Statistical Analysis Method

Time-to-event analysis technique was used to understand time to resolution of colic symptoms, i.e., constant reduction in crying time in infants over 28 days of study. The standard percentage of daily crying time reduction considered to be a clinical response was 50% from baseline. Similarly, responders with 40% and 30% reduction were also assessed.

## 3. Results

**Figure 1:**
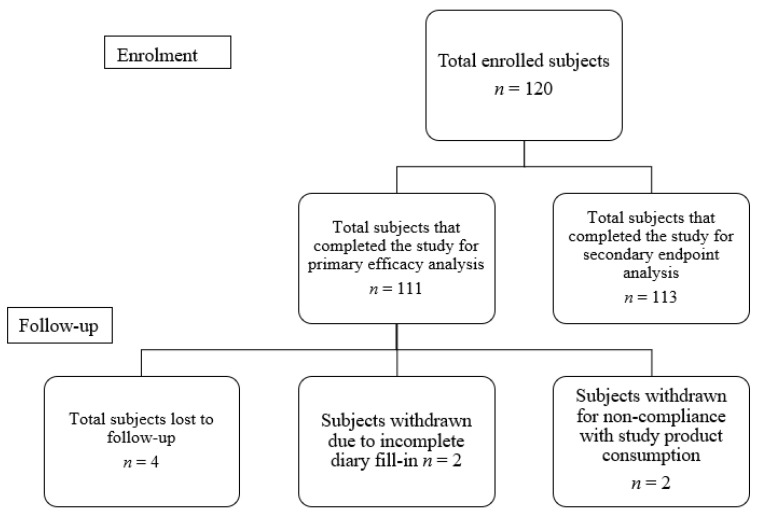
Participant disposition.

A total of 120 infants were recruited, amongst which 53 (44.2%) were female and 67 (55.8%) were male. A total of 112 infants were included in the analysis data set (Figure 1). Eight infants were excluded from primary endpoint analysis due to loss to follow-up (*n* = 4), prescribed antibiotics (*n* = 1) and un-filled daily diaries (*n* = 3). The mean age of all enrolled infants was 56.9 ± 34.2 days. The mean birth weight and body length was 2.8 ± 0.4 kg and 53.0 ± 5.5 cm. The head circumference average was 36.6 ± 2.9 cm.

### 3.1. Primary Outcomes

**Figure 2:**
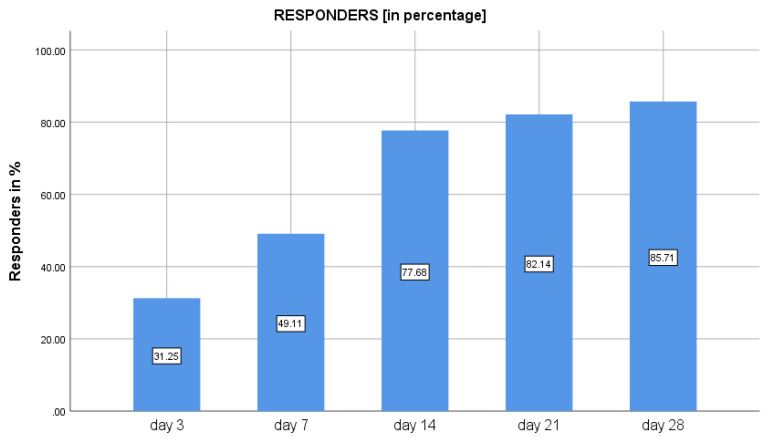
Percentage of responders with 50% reduction in crying time following use of *Lactobacillus reuteri DSM 17938* in infantile colic.

At baseline, the mean crying time was 248.2 ± 101.2 min, which reduced to 171.5 ± 91.2 min on day 3, representing a 25% decrease from the baseline. The reduction in crying time from baseline to day 7, 14, and 21 was 41%, 60%, and 72%, respectively. The decrease in crying time was maintained post-therapy from day 21 to day 28, being 45.7 ± 79.1 min on day 28 (*P* < 0.01). The overall percentage reduction of crying time between day 0 and day 28 was 78.9% (Table 1). The percentages of responders with 50% reduction in crying time compared to baseline were 31.3% (*n* = 35) on day 3, 49% (*n* = 55) on day 7, 77% (*n* = 87) on day 14, 82% (*n* = 92) on day 21, and 85% (*n* = 96) on day 28 (Figure 2).

### 3.2. Secondary Outcomes

**Figure 3:**
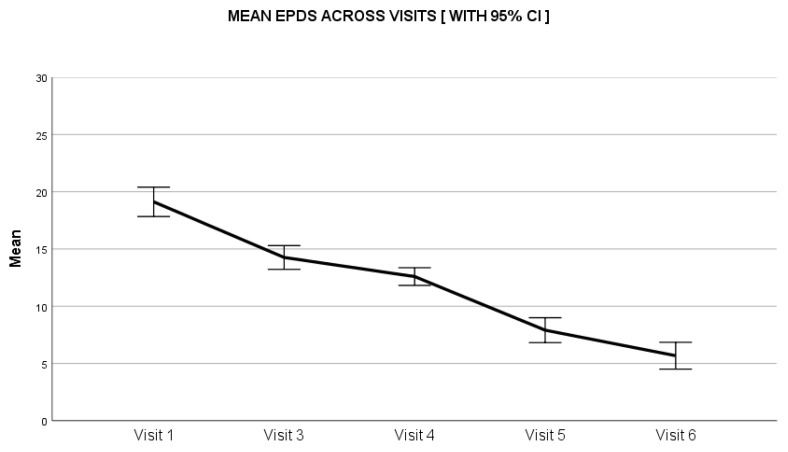
Impact of *Lactobacillus reuteri DSM 17938* administration on analysis of maternal depression score. CI: Confidence Interval.

**Figure 4:**
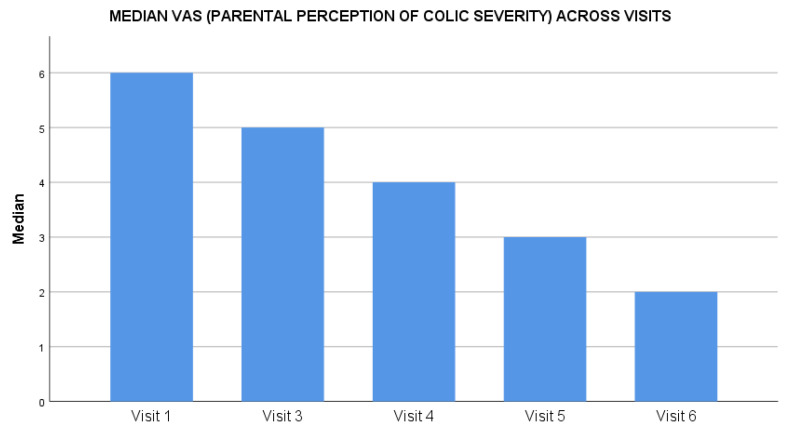
Impact of *Lactobacillus reuteri DSM 17938* administration on parental perception of colic severity during the treatment.

The maternal depression scores assessed through the EPDS scale reduced by 63% from baseline to end of the study. On day 0, the median score was 18 (range: 3–30), which reduced throughout the study duration to a median score of 3 (range: 0–26) at day 28 (Figure 3). The average fussiness per day recorded in the FRS at baseline was 270.9 ± 97.0 min, which reduced to 63.0 ± 63.8 min at the study end visit on day 28, (*P* < 0.01). The average score for amount of fussiness at baseline was 4.5 ± 1.0, which reduced to 1.4 ± 1.1 at the study end visit on day 28, (*P* < 0.01) (Table 2). The intensity of the unexplained fussiness as recorded in the FRS scale had a baseline median score of 5 (range: 2–6) that reduced to 1 (range: 0–3) on day 28. Similarly, the parental perception of colic severity as assessed on a 0 to 10 VAS had a median score of 6 (range: 2–10) at baseline, reducing to a low score of 2 (range: 0–5) on day 28 (Figure 4). The baseline crying time had considerable variability across the four geographical zones, suggestive of a diverse population; however, the reduction in crying time was consistent in all zones during the study period.

#### Results of Exploratory Statistical Analysis

In the time to event analysis, it was observed that the median duration taken to have a constant reduction of 50% in crying time for the infant was 12 days (95% confidence interval: 9.8–14.1). Similarly, the median durations to have constant reduction in crying time to 40% and 30% were 10 days and 7 days, respectively.

## 4. Discussion

Over the decades, various lines of evidence have coalesced to provide strong support on the beneficial effects of different probiotics in conditions causing dysbiosis of the gut microbiota [12]. Alteration of gut microflora has been observed following administration of specific species and strains of probiotics, when administered at adequate dosage [13]. Moreover, data have shown early-age administration of probiotics could provide stimulus to the immature immune system and strengthen the innate and adaptive host responses, thereby creating a balance between the anti- and pro-inflammatory markers [14]. Studies have also shown that infants with infantile colic have high inflammatory markers, such as interleukin-6, interleukin-10, tumor necrosis factor-α, and interferon-γ, which alters gut microbiota [15]. Finally, various studies have shown *L. reuteri DSM 17938* supplementation in colicky infants to reduce the crying time significantly [5,16]. 

The study conducted by Savino et al. reported a significant reduction in the daily crying time at study end by 75% vs. 26% in the Simethicone group [17]. These results were also similar to the study conducted Szajewska et al., where the probiotic group had a crying time reduction of 78% from baseline with 100% treatment success, whereas the placebo group reported a reduction of 50% with a treatment success of 62.5% [16]. Similarly, a study by Indrio et al. proved that prophylactic use of *L reuteri* DSM 17938 during the first three months of life reduced the onset of functional gastrointestinal disorders and reduced private and public costs for the management of this condition [17]. In the meta-analysis performed by Sung, the average crying time reduction on Day 7 was −95.8 min which was similar to −108 min seen in our study and the reduction thereafter was consistent with the results of the meta-analysis [7] In a meta-analysis conducted by Zermiani et al. that included ten clinical trials, the evidence suggests that supplementing *Lactobacillus reuteri* reduced crying time in babies diagnosed with colic [18]. Although this study was not a randomized control trial, the probiotic arm showed a similar reduction in crying time as these studies.

The real-life study described herein found an association with the supplementation of *L. reuteri DSM 17938* and reduction of crying time. The clinical response, defined as reduction of 50% in crying time, was observed in nearly all subjects at study end (with only 15% not showing the response). The time to persistence of reduction of the crying time in all the infants was observed to be rather short (less than 2 weeks) in the time-to-event analysis, which was reported as “satisfactory” by both the parent and the physician. This finding, however, needs to be firmly established by a study in a clinical trial with a control arm. 

Studies have shown that infantile colic and prolonged crying were associated with high maternal depression scores [19]. In our study, the maternal depression scores were reduced by 63%, which was statistically significant from baseline to study end. It was also established that there was a positive association between maternal depression and infant’s crying, which also needs to be re-established in a well-designed controlled trial, considering the importance given to maternal depression in developing countries.

This being an observational study lacked a control arm, thereby excluding the possibility of a maturation effect in the crying time reduction, as seen commonly in disorders with a “resolution with time effect”. For example, in the study conducted by Wolke et al. [20], there was a significant reduction in the duration of fussing/crying at around 8–9 weeks of age. There was also the possibility of a parental bias in reporting modality in our study. Nonetheless, the observed findings of this study, which happens to be the first in infantile colic in India, may enable researchers to further develop well-designed controlled trials to strengthen the overall evidence to support recommendations for use of *L. reuteri DSM 17938* in infantile colic, which has a rising prevalence in India [21].

## 5. Conclusions

This study observed that colicky infants who received *L. reuteri* DSM 17938 showed a significant reduction in crying time over the period, which might be associated with an improvement in maternal depression.

## Figures and Tables

**Table 1:** Analysis of reduction in crying time, *n* = 112.

Days	Crying Time Reduction in Mins	Percentage Reduction	95% Confidence Interval
−1 to 3	−76.6 (113.7)	−25.2 (43.8)	0.9, 1.0
−1 to 7	−108.9 (108.4)	−41.2 (44.0)	1.4, 2.1
−1 to 14	−157.3 (109.8)	−60.8 (42.5)	2.2, 2.9
−1 to 21	−185.8 (116.0)	−72.4 (47.2)	2.7, 3.4
−1 to 28	−202.5 (122.3)	−78.9 (40.3)	2.9, 3.7
21 to 28	−16.6 (45.6)	−37.2 (48.9)	0.1, 0.4

Data are presented as mean (standard deviation).

**Table 2:** Analysis of average crying/fussiness per day and amount of fussiness, *n* = 112.

S. No.	Visit	Mean in Min (Standard Deviation)
Average crying/fussiness per day		
1	1	270.9 (97.0)
2	3	175.4 (87.0)
3	4	139.9 (96.2)
4	5	102.0 (88.4)
5	6	63.0 (63.8)
Visit-wise amount of fussiness		
1	1	4.5 (1.0)
2	3	3.4 (1.2)
3	4	2.8 (1.4)
4	5	2.1 (1.0)
5	6	1.4 (1.1)

## Data Availability

The data presented in this study are available on request from the corresponding author. The data are not publicly available due to confidentiality reasons.
